# The influence of feeding behaviour on growth performance, carcass and meat characteristics of growing pigs

**DOI:** 10.1371/journal.pone.0205572

**Published:** 2018-10-15

**Authors:** Giuseppe Carcò, Luigi Gallo, Mirco Dalla Bona, Maria Angeles Latorre, Manuel Fondevila, Stefano Schiavon

**Affiliations:** 1 Department of Agronomy, Food, Natural Resources, Animals and Environment, University of Padova, Legnaro, Padova, Italy; 2 Department of Animal Production and Food Sciences, IA2 CITA-University of Zaragoza, Zaragoza, Spain; INIA, SPAIN

## Abstract

This study investigated the effect of the feeding behaviour on growth performance, and carcass and meat characteristics of 96 barrows fed *ad libitum* or restrictively with high or low amino acids (AA) diets according to a 2 × 2 factorial design. The feeding behaviour traits were measured with automated feeders. From 86 kg BW, half of the pigs were given feeds with high indispensable (AA) contents, while the other half received feeds with indispensable AA contents reduced by 9% in early finishing (86–118 kg BW) and by 18% in late finishing (118–145 kg BW). Body lipid and protein retentions were estimated from BW and backfat depth measures recorded at the beginning and end of each period. Pigs were slaughtered at 145 kg BW and carcass and meat quality data were recorded. Phenotypic correlations among feeding behaviours, growth performances, and carcass and meat traits were computed from all the data after adjustment for the effects of feeding treatments. As feeding rate was the behavioural trait most highly correlated with performance and carcass traits, the records of each pig were classified into feeding rate tertiles. Then, the data were statistically analysed using a mixed model, which included feed restriction (FR), AA reduction (AAR), the FR × AAR interaction and the feeding rate tertile as fixed factors, and pen as a random factor. Pigs eating faster (52.1 to 118.9 g/min) had significantly greater final body weights (16%), average daily weight gains (27%), estimated protein gains (22%), estimated lipid retention (46%), carcass weights (16%), weights of lean cuts (14%), weights of fat cuts (21%), proportions of fat in the carcass (14%), and 4% lower proportions of carcass lean cuts than pigs eating slowly (12.6 to 38.2 g/min). Manipulating the eating rate, through management or genetic strategies, could affect feed intake and subsequent growth performance, hence carcass quality, but have little influence on feed efficiency.

## Introduction

The availability of automated feeding stations enabled the measurement of the feeding behaviour in growing pigs [1]. Feeding behaviour can be defined by criteria such as the time spent eating per day, feed consumption per day, number of feeding visits, time spent eating per visit and feeding rate [2]. A better knowledge of pig feeding behaviours can help to clarify the role played by factors influencing feed intake, growth performance, feed utilisation efficiency, the quality of the products and the social interrelationships among pigs [3]. Previous studies have explored the phenotypic and genetic relationships among feeding behaviour traits, growth performances and feed efficiency [2,4–7]. Alterations of feeding patterns, such as those imposed by the farmer through feed distribution, have been found to affect feed efficiency and body composition [8–10]. De Haer et al. [2] showed that meal size can negatively affect feed digestibility, and that rate of feed intake and meal size are the factors most commonly associated with growth performance, whereas daily eating time and eating frequency are associated with the residual feed intake. Similarly, Andretta et al. [11] found that feeding rate and number of meals per day were the variables most closely related to performance results. They also found that feed efficiency was negatively correlated with the amount of feed consumed per meal and feeding rate, and that feeding rate was negatively correlated with protein utilisation efficiency.

However, inconsistencies have been found in studies of the mutual influences among feeding behaviour, growth performance, and feed utilisation efficiency [3], which may be due to the very large variability among individuals with respect to these behavioural traits. In general, among-pig variation in behavioural traits is much greater than feed intake variation [12]. A useful way to interpret this huge variability is to consider feeding behaviour as a flexible strategy the pig follows to reach its desired feed intake when kept in a given social and productive environment [1, 12–14]. The desired feed intake, which is the amount of feed required for maintenance and growth, mainly depends on the pig’s genotype and physiological state [15,16], although nutrient imbalances [17,18], and climatic [19,20] and social [4] conditions may also influence the nutritional motivation of the pigs and their desired, and hence actual, feed intake. De Haer et al. [2] found that pigs with different nutritional motivations and feed intake patterns would also have different carcass and meat quality characteristics. In that study, the pigs with the lowest rate of feed intake and the lowest feed consumption per meal had the lowest daily weight gain and the highest estimated carcass lean percentage; the authors suggest that these pigs may have been the subordinate ones in the pen, chased away from the mangers. As a consequence, pigs with the fastest feeding rates or highest feed consumption per visit might also be those with the greatest feed intake, growth rates and carcass fatness, which would in turn affect the lipid content and hence quality of the meat. However, very few studies have examined the effect of the feeding behaviour on carcass traits and meat quality.

Thus, the aim of the current paper is to explore the influence of feeding behaviour on growth performance, carcass and meat characteristics of pigs, using data collected from a previous experiment.

## Material and methods

### Pigs and experimental design

All experimental procedures were reviewed and approved by the University of Padova’s Ethical Committee for the Care and Use of Experimental Animals (Prot. #147683). The data were taken from a previous experiment aimed at investigating the influence of mild restrictions to the feed allowance and dietary amino acid content on the growth performance [17] and feeding behaviour of growing pigs [12].

Briefly, the experiment involved 96 Topigs Talent × PIC barrows born within the same week. They arrived at the experimental station of the University of Padova at the end of February and were slaughtered at the end of June, thereby avoiding hot ambient summer temperatures. The average temperature in the housing rooms ranged from 20 to 25°C, from the start to the end of the trial. The pigs were allotted to 8 pens (5.8 × 3.8 m), at an average body weight (BW) of 35.8 ± 2.82 kg, with 12 pigs/pen. Each pen was equipped with an automated feeding station (Compident Pig—MLP, Schauer Agrotronic, Austria). After an acclimation period of 12 days, 6 pigs in each pen were fed *ad libitum* (AL), while the other 6 were subjected to a moderate restricted feeding regime (RF) from 47 to 145 kg BW. Each pig of the RF group was allowed to consume, as a maximum, the daily feed amount suggested by the breeding company for Topigs Talent barrows [21], and daily feed allowance ranged between 2.15 and 2.80 kg at the start and the end of the trial, respectively. The RF plane aimed to prevent excessive feed consumption by the greedier pigs, and resulted in a 7% lower average feed intake with respect to AL pigs, according to Schiavon et al. [17]. From 86 kg BW upwards, the pigs of 4 pens were given feeds with high indispensable AA contents (HAA), in slight excess of NRC recommendations [22], while the pigs of the other 4 pens were given feeds with indispensable AA (LAA) reduced by 9% in early finishing (86–118 kg BW) and by 18% in late finishing (118–145 kg BW), with respect to the HAA diet. The dietary composition is given in Table 1, and major details about the experimental conditions are given in Schiavon et al. [17].

**Table 1:** Chemical composition (g/kg) and energy content (MJ/kg) of the diets.

Item	Growing (47–86 kg BW)	Early finishing (86–118 kg BW)		Late finishing (118–145 kg BW)	
		High amino acid (HAA)	Low amino acid (LAA)	High amino acid (HAA)	Low amino acid (LAA)
Analyzed composition^1^					
Dry Matter	893	891	891	895	894
Crude Protein (N × 6.25)	163	159	141	161	133
Lysine	10.3	9.3	8.5	8.8	7.2
Methionine	3.5	3.1	2.7	3.0	2.5
Threonine	7.0	6.3	5.9	6.4	5.1
Tryptophan	2.2	2.0	1.8	1.9	1.6
Starch	387	440	454	421	454
NDF	120	111	111	123	130
Ether Extract	46	42	45	44	42
Ash	41	42	40	42	41
Calculated composition^2^					
Dry Matter	879	879	878	878	877
Net Energy	9.9	9.8	9.8	9.7	9.8
Crude Protein (CP)	161	158	143	155	126
SID Lysine^3^, g/kg CP	56	51	52	47	48
SID Methionine^3^, g/kg CP	20	17	17	17	17
SID Threonine^3^, g/kg CP	37	34	34	34	33
SID Tryptophan^3^, g/kg CP	10	10	10	10	10

^1^Analitical results as a mean from 3 independent replications.

^2^ Computed from the ingredient composition according to NRC [22].

^3^ SID: standardized ileal digestible amino acid content.

The feeding stations of pens allowed the pigs access to the feed throughout the whole day. The AL pigs were able to access the station and eat as much as they wished all day, whereas the RF pigs had 24 h access to the station, but were allowed to eat up to 0.33, up to 0.66 and up to 1.00 portions of the daily planned feed ration during the 0.01–8.00 h, 8.01–16.00 h, and 16.01–24.00 h time intervals, respectively. Lateral barriers restrained competition among the pigs during eating. A gate placed in front of the trough was opened only after the pig identification and avoided that other pigs could steal the feed. The date and time of feeding, the time spent eating and the weights of the feed consumed and left over by each individual pig were recorded. The leftovers were weighed and assigned to the next pig visiting the station. All the pigs had free access to a nipple drinker placed in each pen.

Individual BW was measured weekly using an electronic scale, and backfat depth (BF) was measured every two weeks with an A-mode ultrasonic device (Renco Lean-Meater series 12, Renco Corporation, Minneapolis, USA) from 86 kg BW upwards. The BF measure was taken above the last rib at approximately 5.5–8.0 cm from the midline, the distance increasing with increasing BW [23].

### Slaughter and assessment of carcass data and meat quality

All pigs were slaughtered on the same day in one batch after 24 h of fasting. They were stunned with carbon dioxide, and killed by exsanguination after cutting the jugular vein, according to standard slaughter house procedures. Carcasses were scalded, de-haired, eviscerated and split down the midline, according to commercial slaughtering procedures. Individual hot carcass weights were recorded and the dressing percentages calculated. Carcass lean percentage [24–25] was calculated from BF and loin depth measurements taken on the left side of each carcass between the 3^rd^ and 4^th^ ribs 8 cm off the midline using a FOM (Fat-O-Meat’er, Carometec, Soeborg, Denmark).

Hot carcasses were processed according to the standard commercial procedure to obtain the main lean cuts (loin with ribs, neck with bones but without skin and subcutaneous tissues, shoulder with bones and skin, and ham) and fat (backfat and belly) primal cuts, which were separately weighed.

A sample of the *Longissimus lumborum* (LL) including the last two lumbar vertebrae was collected from the left loin of each carcass, and each sample placed in individual plastic bags, refrigerated for 24 h, then vacuum-packed at -20 °C for subsequent analyses. After 24 h of chilling, the thighs were deboned, and then weighed.

The LL samples were thawed in vacuum-packaged bags for 24 h at 4 °C, then removed from the packaging and blotted for 15 min and weighed. Thawing losses were calculated as the difference in weight between the fresh and thawed samples expressed as a percentage of the initial fresh weight.

Cooking losses were determined on a subsample of LL 2.5 cm in thickness, which was weighed, sealed in a plastic bag, cooked in a water bath at 75° C until it reached a core temperature of 70° C, then cooled to room temperature, blotted and weighed. Cooking loss percentages were calculated by dividing the difference between the pre- and post-cooked weights by the pre-cooked weight.

Shear force was measured on five cylindrical cores 1.00 cm in diameter taken from the same cooked sample sheared perpendicularly with a Lloyd (Bognor Regis, UK) LS 5 series Warner-Bratzler shearing device (shearing speed 2 mm s^-1^) managed by the NEXIGEN Plus 3 software. The measurements from each sample were averaged before statistical analyses.

Another subsample of LL was ground, mixed and homogenised for 10 s at 4500 g in a Grindomix GM200 (Retsch, Haan, Düsseldorf, Germany) then analysed in duplicate for moisture (# 950.46), protein (# 981.10), lipids (#991.36) and ash (# 920.153) [26].

### Data editing

During the experiment, 4 pigs died or were discarded due to illness or injury and their data were removed from the database, thus the final dataset consisted of data from 92 pigs. Six behavioural traits were analysed from the data recorded by the feeding stations after excluding visits where feed consumption was zero (Table 2). The main details are given in Carcò et al. [12].

**Table 2:** Individual feeding behaviour parameters and the criteria used to compute them.

Parameter	Criterion
Feed intake (g/d)	feed consumed in a given day by a pig
Time spent eating (min/d)	total duration of the visits in a given day by a pig
Feeding visits (n/d)	visit with feed intake > 0 g by a pig
Feed intake per visit (g/visit)	average amount of feed consumed per visit by a pig
Feeding time per visit (min/visit)	the time spent eating per visit by a pig
Feeding rate, g/min	feed intake per visit / visit duration by a pig

Protein (Pr) and lipid (Lr) retentions were estimated from BW and ultrasound BF measurements recorded at the beginning and end of the trial, as described in Schiavon et al. (2018) [17]. Residual metabolizable energy intake (REI, MJ/d) was determined for each pig as the difference between metabolizable energy (ME) intake and ME used for maintenance (MEm) and growth (MEg). ME intake was calculated as the total feed intake × ME of the diet; MEm as 0.845 MJ × the average BW of the period^0.6^; and MEg as 44.35 × Pr + 52.30 × Lr, in accordance with the NRC [22].

### Statistical analysis

Individual day-by-day patterns of variation in each behavioural trait were averaged for all the pigs of the experiment, edited, plotted and regressed against the days on feed in a spreadsheet. The SAS PROC CORR [27] was carried out to investigate the correlations between the behavioural traits and the days on feed, in order to test the magnitude and the significance of each trend.

The data regarding feeding behaviour and growth performance averaged by pig, and carcass and meat quality traits were analysed for deviation from normality in SAS [27].

To adjust the data for the effects of the feeding treatments, a preliminary analysis of the individual means of each trait was carried out using SAS PROC GLM [15] and a model that included feed restriction (FR), amino acid restriction (AAR), and the FR × AAR interaction as fixed effects, and pen within AAR as a random effect. The residuals were analysed using SAS PROC CORR [27], and the partial correlation coefficients among variables were computed. As feeding rate was found to be the behavioural trait most frequently and highly correlated with the carcass characteristics, the records of each pig were classified according to feeding rate tertiles computed on the residuals of the previous model. Data were analysed by SAS PROC MIXED [27] using the model described above with the further inclusion of the feeding rate tertile as a fixed factor and its interaction with FR and AAR. As these interactions were never significant, they were excluded from the final model. The pig was considered to be the experimental unit to test the influence of the feeding rate. Two of the three degrees of freedom of the feeding rate tertile were used to evaluate the significance of the linear and quadratic components.

## Results

### Patterns in the feeding behaviour traits

Readers are referred to Carcò et al. [12] and Schiavon et al. [17] for details on the effect of feed allowance and AA level on feeding behaviour, growth performance and carcass and meat quality.

**Fig 1:**
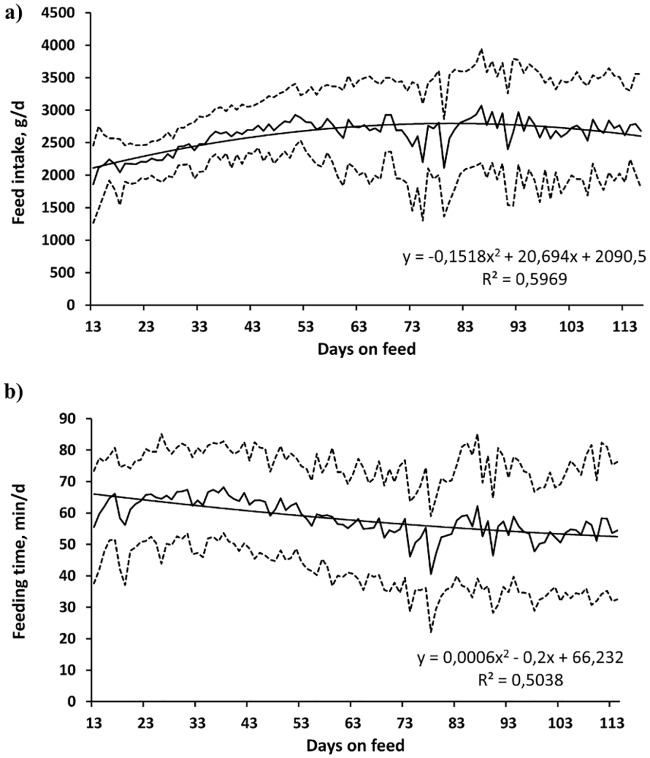
Growing pigs’ individual patterns in feed intake (A) and time spent eating (B) with increasing days on feeding regimes (n = 92, Mean = thick line; mean ± standard deviation = dotted line, trend = thin line; the experiment started on the 13^th^ day after the pigs’ arrival).

**Fig 2:**
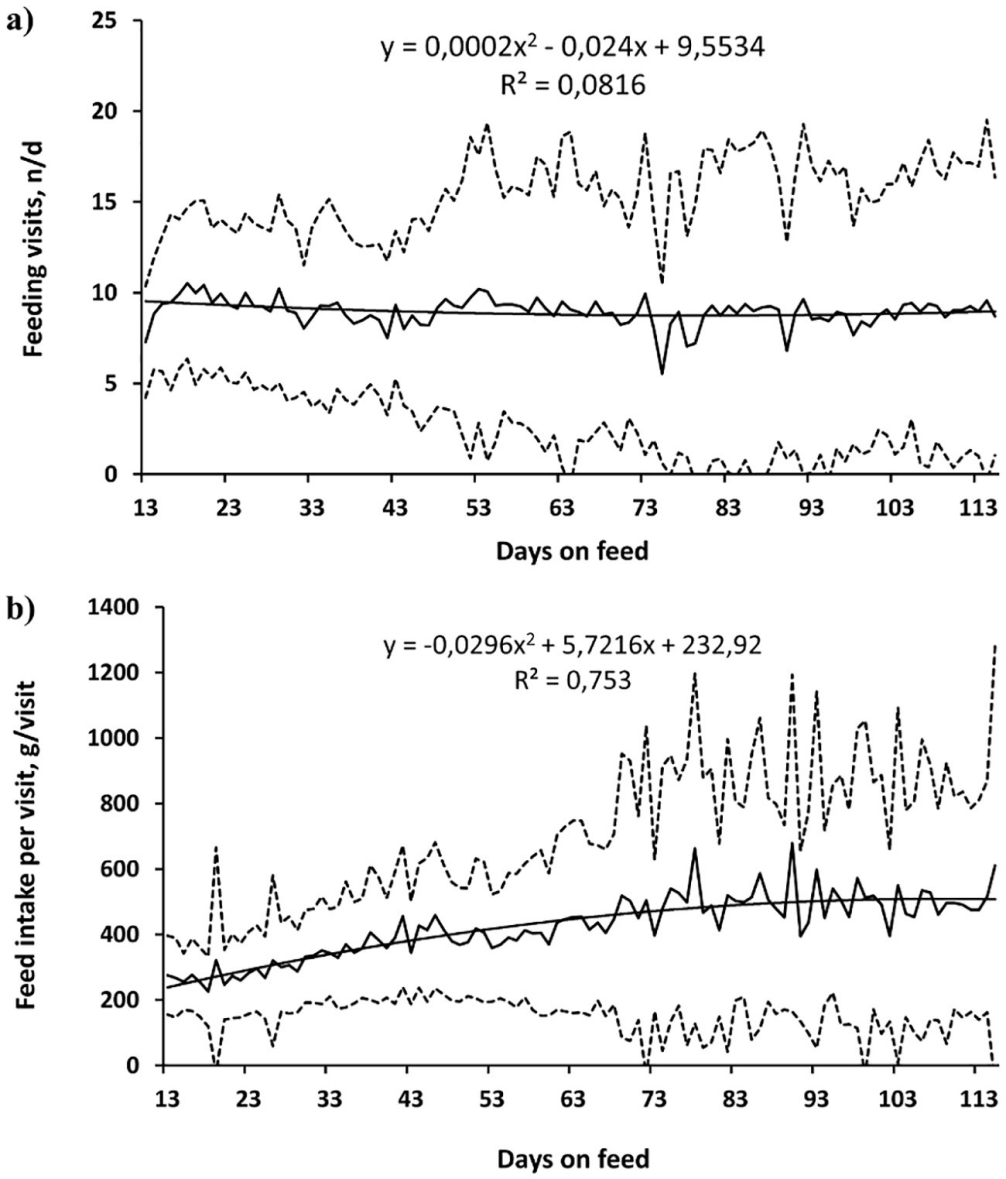
Growing pigs’ individual patterns in the number of feeding visits (A) and feed intake per visit (B) with increasing days on feeding regimes (n = 92, Mean = thick line; mean ± standard deviation = dotted line, trend = thin line; the experiment started on the 13^th^ day after the pigs’ arrival).

**Fig 3:**
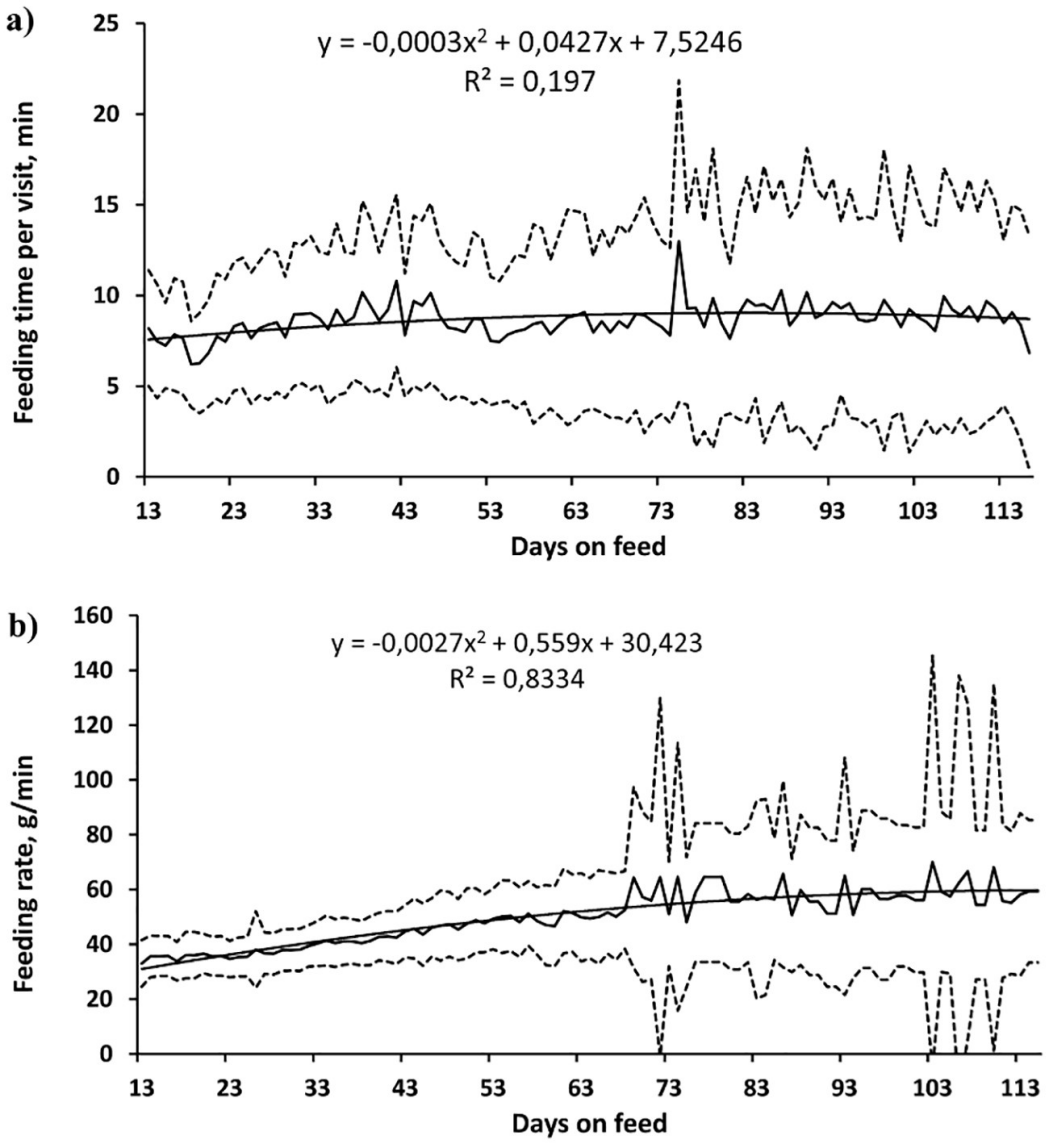
Growing pigs’ individual patterns in duration of feeding time per visit (A) and feeding rate (B) with increasing days on feeding regime (n = 92, Mean = thick line; mean ± standard deviation = dotted line, trend = thin line; the experiment started on the 13^th^ day after the pigs’ arrival).

The average daily feed intake increased and the time spent eating decreased with the number of days on feed (R^2^ = 0.60, *P* < 0.001, Fig 1A; and R^2^ = 0.50, *P* < 0.001, Fig 1B, respectively), and there were only small variations in the number of visits to the manger (R^2^ = 0.08, *P* = 0.028, Fig 2A). The standard deviation among individuals with respect to all these variables was large. The amount of feed consumed per visit increased quadratically (R^2^ = 0.75, *P* < 0.001, Fig 2B) and there was a small change in the time spent eating per visit (R^2^ = 0.20, *P* < 0.001, Fig 3A) with increasing days on feed. The feeding rate changed quadratically (R^2^ = 0.83, *P* < 0.001, Fig 3B) and the standard deviation, in the order of 50% of the mean, increased notably towards the end of the period of observation.

### Partial correlations between feeding behaviour and performance traits

The partial phenotypic correlations among the feeding behaviour traits are given in S1 Table.

Daily feed intake was positively correlated with final BW (r = 0.821, *P* < 0.001), growth rate (r = 0.847, *P* < 0.001), Pr (r = 0.230, *P* < 0.05) and REI (r = 0.267, *P* < 0.05) (Table 3). Time spent eating was negatively correlated with final BW (r = -0.247, *P* < 0.05), growth rate (r = -0.253, *P* < 0.01), the gain:feed ratio (r = -0.224, *P* < 0.05) and Lr (r = -0.360, *P* < 0.001), but it did not affect feed intake and REI.

**Table 3:** Partial correlations between feeding behaviour traits and growth performance (n = 92)^1^.

Item	Feed intake	Time spent eating	Feeding visits	Feed intake per visit	Feeding time per visit	Feeding rate
Initial body weight, kg	-0.083	0.019	-0.110	0.046	0.140	-0.054
Final body weight, kg	0.821***	-0.247*	-0.106	0.268**	-0.121	0.522***
Feed intake, kg/d	-	-0.143	-0.003	0.203*	-0.190	0.506***
Growth rate, kg/d	0.847***	-0.253**	-0.067	0.251*	-0.170	0.539***
Protein retention (Pr), g/d^2^	0.230*	0.167	-0.044	0.147	0.173	0.408***
Lipid retention (Lr), g/d^3^	-0.152	-0.360***	-0.069	0.260*	-0.070	0.430***
Feed efficiency (Gain:feed)	-0.096	-0.224*	-0.113	0.121	0.001	0.150
Residual energy intake^4^	0.267*	0.133	0.174	-0.168	-0.179	-0.001

^1^ *, **, and *** stand for P < 0.05, P < 0.01 and P < 0.001.

^2^ Estimated from body protein mass changes (kg), from 47 to 145 kg BW. Body protein mass was estimated as 0.1353 × FFEBW^1.1175^ (NRC, 2012), where FFEBW is the fat free empty body weight.

^3^ Estimated from body lipid mass changes (kg), from 47 to 145 kg BW. Body lipid mass was estimated from backfat and body weight (BW) according to Kloareg et al., [11].

^4^ Computed as: metabolizable energy (ME) intake − (ME req. for maintenance + ME req. for growth), where ME req. for maintenance = BW^0.60^ × 0.845 MJ, and ME req. for growth = 44.35 × Pr + 52.30 × Lr [10].

The number of feeding visits and the feeding time per visit were not correlated with growth performance and feed efficiency, whereas feed intake per visit was positively related to final BW (r = 0.268, *P* <0.01), growth rate (r = 0.251, *P* < 0.05), Lr (r = 0.260, *P* < 0.05) and daily feed intake (r = 0.203, *P* <0.05). The time spent per visit did not affect growth performance.

Feeding rate was the behavioural trait most highly correlated with final BW (r = 0.522, *P* < 0.001), growth rate (r = 0.539, *P* < 0.001), and both Pr (r = 0.408, *P* < 0.001) and Lr (r = 0.430, *P* < 0.001). Also, feeding rate was positively correlated with daily feed intake (r = 0.506, *P* < 0.001), but not with feed or energy efficiency traits.

### Partial correlations between feeding behaviour, carcass traits and meat quality traits

Feed intake and feeding rate were the variables most highly correlated with the various carcass traits (Table 4). Feed intake was positively related to carcass weight (r = 0.840, *P* < 0.001), backfat thickness (r = 0.595, *P <* 0.001), the weights of all the untrimmed lean and fat cuts (*P* < 0.001) and the proportions of carcass fat components (r = 0.663, *P* < 0.001), but was negatively correlated with the proportions of lean cuts (r = -0.677, *P* < 0.001). Feed intake was also negatively related to the moisture content of the muscle (r = -0.249, *P* < 0.05) and positively related to the lipids content (r = 0.265, *P* < 0.01).

**Table 4:** Partial correlations between feeding behaviour traits and carcass and meat quality (n = 92)^1^.

Item	Feed intake	Time spent eating	Feeding visits	Feed intake per visit	Feeding time per visit	Feeding rate
Carcass weight, kg	0.840***	-0.256*	-0.102	0.272**	-0.124	0.535***
Carcass yield, %	0.111	-0.057	0.002	0.039	-0.017	0.079
Backfat thickness^2^, mm	0.595***	-0.050	0.064	0.093	-0.098	0.269*
Loin depth^2^, mm	0.039	-0.012	-0.010	0.076	0.033	-0.028
Lean percentage (FOM)^3^, %	-0.071	0.056	0.043	-0.052	-0.002	-0.061
Main untrimmed lean and fat cuts, kg:						
- loin with ribs	0.543***	-0.222*	-0.009	0.163	-0.136	0.387***
- neck	0.476***	0.118	-0.089	0.171	-0.011	0.249*
- shoulder	0.465***	-0.123	-0.045	0.114	-0.094	0.280**
- ham	0.625***	-0.305**	-0.219*	0.320**	-0.029	0.485***
- deboned ham	0.532***	-0.285**	-0.130	0.259*	-0.059	0.439***
- backfat	0.812***	-0.137	0.016	0.161	-0.175	0.452***
- belly	0.771***	-0.306**	0.096	0.288**	-0.121	0.557***
- total main lean cuts	0.613***	-0.256*	-0.116	0.225*	-0.101	0.440***
- total main fat cuts	0.857***	-0.230*	-0.037	0.236*	-0.163	0.539***
Yield of untrimmed lean and fat cuts, % of carcass:						
- total lean	-0.677***	0.086	0.002	-0.177	0.085	-0.344**
- total fat	0.663***	-0.165	0.027	0.158	-0.158	0.419***
Yield of deboned ham, % of untrimmed ham	-0.293**	0.086	0.239*	-0.183	-0.066	-0.168
Longissimus lumborum (LL) muscle composition, %						
- moisture	-0.249*	0.143	0.303**	-0.326**	-0.175	-0.191
- protein	-0.082	-0.047	-0.307**	0.204	0.268**	0.018
- lipids	0.265**	-0.099	-0.085	0.167	-0.004	0.157
- ash	0.029	0.033	-0.066	0.018	0.037	0.019
Water holding capacity of LL, %						
- thawing loss	0.099	-0.026	0.093	-0.055	-0.114	0.032
- cooking loss	-0.197	0.016	0.031	-0.046	0.008	-0.087
Warner-Bratzler shear force of LL, kg	-0.090	-0.012	-0.204	0.082	0.123	-0.047

^1^ *, **, and *** stand for P < 0.05, P < 0.01 and P < 0.001.

^2^ Assessed with a Fat-O-Meat’er between the third to fourth last ribs at 8 cm off the carcass midline.

^3^ Calculated from backfat thickness and loin depth taken between the third to fourth last ribs at 8 cm off the carcass midline.

Feeding rate was positively related to carcass weight (r = 0.535, *P* < 0.001), BF (r = 0.269, *P* < 0.05), the weights of all the separated lean and fat cuts (*P* < 0.001), and the proportions of carcass fatty tissues (r = 0.419, *P* < 0.001), and negatively related to the proportions of carcass lean cuts (r = -0.344, *P* < 0.01). We found no significant relation between feeding rate and the chemical and physical characteristics of the LL muscle.

Time spent eating was negatively related to carcass weight (r = -0.256, *P* < 0.05) and the weights of some lean and fat cuts. Feed intake per visit also was also related to carcass characteristics as it was positively related to carcass weight (r = 0.272, *P* < 0.01), the weights of some cuts, like the ham (r = 0.32, *P* < 0.01), belly (r = 0.288, *P* < 0.01) and total lean and fat cuts (*P* < 0.05).

The number of visits was poorly related to carcass and meat characteristics, although this trait was positively related to the moisture content of the *Longissimus lumborum* muscle (r = 0.303, *P* < 0.01) and negatively related to its protein content (r = 0.307, *P* < 0.01). The average time spent eating per visit was positively related only to the protein content of the *Longissimus lumborum* muscle (r = 0.268, *P* < 0.01).

### Feeding rate, growth performance, carcass traits and meat quality

The distribution of RF and AL and HAA and LAA pigs in the classes of feeding rate was homogenous. The class of feeding rate had a linear influence on final BW (*P* < 0.001), growth rate (*P* < 0.001), Pr (*P* < 0.001), Lr (*P* < 0.001) and feed intake (*P* < 0.001) (Table 5), with values that increased moving from the first (12.6 to 38.2 g/min), to the second (38.3 to 51.6 g/min) and the third (52.1 to 118.9 g/min) tertiles. Moreover, it was consistently linearly related to carcass weight (*P* < 0.001), but not to carcass yield, BF thickness, loin depth and lean percentage (Table 6). Feeding rate was also positively linearly related to the weights of all the lean and fat cuts, the linear decrease in the carcass lean percentage (*P* = 0.014), and the linear increase in carcass fat content (*P* < 0.001). Since no correlation were found between feeding rate and meat quality traits, the class of feeding rate had no influence on the meat quality parameters.

**Table 5:** Influence of feeding rate on the growth performance of barrows (n = 92).

Item	Class of feeding rate				*P values*^1^	
	12.6 to 38.2 g/min	38.3 to 51.6 g/min	52.1 to 118.9 g/min	SEM^2^	L	Q
Initial body weight, kg	48.5	45.7	47.3	1.19	0.46	0.14
Final body weight, kg	131.6	146.1	152.4	2.91	<0.001	0.24
Feed intake, kg/d	2.296	2.707	2.845	0.07	<0.001	0.11
Growth rate, kg/d	0.807	0.975	1.021	0.03	<0.001	0.07
Gain:feed ratio	0.352	0.360	0.360	0.01	0.39	0.65
Residual energy intake^3^	1.97	1.98	2.63	0.53	0.39	0.62
Protein retention (Pr), g/d^4^	143	164	174	5.00	<0.001	0.38
Lipid retention (Lr), g/d^5^	192	270	280	20.0	<0.001	0.08

^1^ L = linear component, Q = quadratic component.

^2^ Standard error of the means.

^3^ Computed as: metabolizable energy (ME) intake − (ME requirement for maintenance + ME requirement for growth), where ME req. for maintenance = BW^0.60^ × 0.845 MJ (NRC, 2012) and ME req. for growth = 44.35 × Pr + 52.30 × Lr [10].

^4^ Computed from body protein mass changes (kg), from 47 to 145 kg BW. Body protein mass was calculated as 0.1353 × fat free empty body weight^1.1175^ [10].

^5^ Computed from body lipid mass changes (kg), from 47 to 145 kg BW. Body lipid mass was estimated from backfat thickness and body weight [11].

**Table 6:** Influence of feeding rate on the carcass and meat quality traits of barrows (n = 92).

Item	Class of feeding rate				*P values*^1^	
	12.6 to 38.2 g/min	38.3 to 51.6 g/min	52.1 to 118.9 g/min	SEM^2^	L	Q
Carcass weight, kg	105.1	117.2	122.5	2.33	<0.001	0.25
Carcass yield, %	0.80	0.80	0.81	0.01	0.45	0.93
Backfat thickness^3^, mm	16.5	20.9	20.4	1.41	0.05	0.16
Loin depth^3^, mm	62.4	63.9	61.5	2.92	0.82	0.56
Lean percentage (FOM)^4^, %	56.6	53.9	54.3	2.26	0.42	0.51
Main untrimmed lean and fat cuts, kg:						
- loin with ribs	18.4	19.5	20.4	0.44	0.002	0.79
- neck	7.69	8.10	8.42	0.20	0.013	0.86
- shoulder	15.9	17.1	17.4	0.38	0.007	0.33
- ham	28.2	31.3	32.4	0.61	<0.001	0.17
- deboned ham	17.9	19.4	20.1	0.38	<0.001	0.42
- backfat	7.03	8.78	9.81	0.52	<0.001	0.56
- belly	11.5	13.7	14.7	0.39	<0.001	0.27
- total main lean cuts	68.8	76.1	78.6	1.41	<0.001	0.28
- total main fat cuts	18.5	22.4	24.5	0.82	<0.001	0.37
Yield of untrimmed lean and fat cuts, % of carcass:						
- total lean	66.5	64.9	64.1	0.68	0.014	0.58
- total fat	17.6	19.2	20.1	0.49	<0.001	0.51
Yield of deboned ham, % of untrimmed ham	63.4	61.8	62.0	0.55	0.07	0.20
Longissimus lumborum (LL) muscle composition, %						
- moisture	71.6	70.8	70.9	0.36	0.15	0.29
- protein	23.5	23.7	23.4	0.24	0.87	0.34
- lipids	3.74	4.31	4.53	0.40	0.18	0.72
- ash	1.18	1.18	1.18	0.01	0.90	0.98
Water holding capacity of LL, %						
- thawing loss	10.2	9.59	11.6	1.01	0.33	0.30
- cooking loss	31.3	29.4	30.2	0.57	0.19	0.07
Warner-Bratzler shear force of LL, kg	2.19	2.41	2.16	0.124	0.84	0.12

^1^ L = linear component, Q = quadratic component.

^2^ Standard error of the means.

^3^ Assessed with a Fat-O-Meat’er between the third to fourth last ribs at 8 cm off the carcass midline.

^4^ Calculated from backfat thickness and loin depth taken between the third to fourth last ribs at 8 cm off the carcass midline [12,13].

## Discussion

### Feeding rate, feed intake and growth performance

The current literature provides evidence that feeding rate could reflect the pig’s feeding motivation, with faster rates associated with greater feeding motivation [3,12–13].

Firstly, greater feeding motivation may reflect a greater desire for the nutrients required for maintenance and for protein and lipid growth [13]. This would, in turn, result in greater feed intake, and different carcass and meat characteristics. In the companion paper to the current study, a reduction in the essential amino acid content of the diet was found to increase the feeding rate, feed intake, growth rate, carcass yield and carcass fat content [17]. Labroue et al. [5] found that the feeding rate had a high genetic correlation with daily feed intake (around 0.5) and average daily gain (around 0.4). In the current study, feeding rate was the variable most highly correlated with the estimated daily gains in protein and lipids, while the variation in feeding rate only partially explained the variation in daily feed intake (r = 0.51), due to the contextual variation in the number and duration of the feeding visits. The phenotypic relationship between feeding rate and daily feed intake was slightly stronger than that observed by de Haer & Merks [28], Labroue et al. [29] and Hyun et al. [30] in pigs penned in groups (r values ranging 0.17 to 0.41), but lower than that observed by de Haer & Merks [28] in individually penned pigs (r = 0.81).

Secondly, pigs have frequently been found to respond to a feeding constraint by increasing their feeding rate [31–32]. For example, recent experiments found that a feeding restriction increased the rate of feed consumption [12,17]. In this regard, Nielsen [13] suggested that feeding rate could be used as an indicator of social constraint. Young & Lawrence [33] found that where there was strong competition among pigs for feed, there was an increase in the feeding rate and number of visits, with a consequent reduction in feed consumption per visit. Similarly, pigs housed in groups notably increased their feeding rate compared with pigs housed individually [4]. Nielsen [13] also reported that in a given social context the pig’s feeding behaviour is influenced by the desire to eat at the same time as its conspecifics.

For the current study, the data presented in the companion papers of Schiavon et al. [17] and Carcò et al. [12] were statistically adjusted for the effects of the experimental treatments. Nevertheless, there was wide among-pig variation in feeding rate, which was about three times greater for the pigs in the third tertile than those in the first. The pigs in the third tertile—those that ate faster—had 16% heavier final body weights, 27% greater average daily weight gains, 22% greater estimated protein gains, and 46% greater estimated lipid retention than the pigs in the first tertile (13 to 38 g/min). The magnitude of these differences may be due to individual variations in the desired nutrient intake, which might be reflected in different body constituent growth rates, and/or to the social hierarchy, which impacts on the feeding strategy followed by each pig to reach its preferred, or constrained, feed intake. It should be borne in mind that in the current study the feeding station in each pen gave access to only one pig at a time, and it is not clear whether this restriction had an impact on the feeding motivation of the other pigs accessing it later. The role of the social environment in pig feeding behaviour and its impact on performance needs to be further clarified.

### Feeding rate, and carcass and meat characteristics

Surprisingly, we found few studies on relationships between carcass and meat characteristics and feeding behaviour traits, despite the economic importance of this issue. De Haer et al. [2] found that pigs that consumed larger amounts of feed per visit and at faster rates of eating exhibited greater growth rates, thicker carcass backfat depths and lower lean percentages. Colpoys et al. [3] did not find any significant correlations among feeding rate, daily feed intake, daily weight gain, and tissue accretion of protein, lean and fat estimated with dual X-ray tomography, but they only studied a small number of gilts fed either *ad libitum* or twice a day.

In the current study, the feeding rate had a strong influence on carcass characteristics. Compared to the group of pigs eating slowly, the pigs of the third tertile, eating at a rate of 52–119 g/min, had greater carcass weight (16%, without change in carcass yields), weight of lean cuts (14%), weight of fat cuts (21%), proportion of fat in carcass (14%), and a corresponding 4% decrease of the proportion of carcass lean cuts. Data measured on the carcass were quantitatively consistent with the *in vivo* estimates of Pr and Lr. Interestingly, the feeding rate had almost no effect on feed efficiency and meat quality traits. These results are similar to the findings of de Haer et al. [2], and to those of Rauw et al. [7] who found that the pigs that ate faster also ate more and grew faster and became fatter, but with the same residual feed intake. In some productive situations, strategies to increase the feeding rate may be based on dietary imbalances in some nutrients. For example, Schiavon et al. [17] found that a small reduction in dietary amino acid stimulated the pigs to increase their feed intake to compensate for this reduction, but in doing so they consumed more energy, and increased growth and fat accretion. The dataset we used in the current study was not suitable for estimating the heritability and the genetic correlations. However, previous studies of Von Felde et al. [34] and Schulze et al. [35] have found high heritability (h^2^ = 0.42–0.51) for several feeding behaviour traits. This information is needed to assess whether the rate of feed intake, or any other feeding behaviour trait, could be included in the selection objectives.

## Conclusion

The results of the current study support the idea that the feeding rate reflects the pig’s feeding motivation, with faster rates associated with increased feeding motivation. Feeding behaviour traits were highly correlated with growth performance and carcass quality. Namely, growth rate, final body weight and carcass traits were positively related to feed intake and feeding rate, but negatively related to the time spent eating. Among the behavioural traits, feeding rate was the one most frequently and highly correlated with daily feed intake, growth rate, protein and fat retention and many carcass traits. Manipulating the eating rate would affect feed intake and subsequently growth performance and carcass quality, but would have little influence on feed efficiency.

## Supporting information

S1 Table Partial correlations among feeding behavior traits (n = 92). (DOCX)

## References

[1] Maselyne J, Saeys W, Van Nuffel A. Review: Quantifying animal feeding behaviour with a focus on pigs. Physiol. Behav. 2015, 138: 37–51. 10.1016/j.physbeh.2014.09.012 25447478

[2] de Haer LCM, Luiting P, Aarts HLM. Relationship between individual (residual) feed intake and feed intake pattern in group housed growing pigs. Livest. Prod. Sci. 1993, 36: 233–253.

[3] Colpoys JD, Johnson AK, Gabler NK. Daily feeding regimen impacts pig growth and behavior. Physiol. & Behav. 2016, 159: 27–32. 26957037 10.1016/j.physbeh.2016.03.003

[4] de Haer LCM, de Vries AG. Feed intake patterns of and feed digestibility in growing pigs housed individually or in groups. Livest. Prod. Sci. 1993, 33: 277–292.

[5] Labroue F, Guéblez R, Sellier P, Meunier-Salaün MC. Feeding behaviour of group-housed Large White and Landrace pigs in French central test stations. Livest. Prod. Sci. 1994, 40: 303–312.

[6] Meunier-Salaun MC, Guérin C, Billon Y, Sellier P, Noblet J, Gilbert H. Divergent selection for residual feed intake in group-housed growing pigs: characteristics of physical and behavioural acitivity according to line and sex. Anim. 2014, 8: 1898–1906. 10.1017/S1751731114001839 25322792

[7] Rauw WM, Soler J, Tibau J, Reixach J, Raya LG. Feeding time and feeding rate and its relationship with feed intake, feed efficiency, growth rate, and rate of fat deposition in growing Duroc barrows. Am. Soc. Anim. Sci.2006, 84: 3404–3409. 10.2527/jas.2006-209 17093234

[8] Le Naou T, Le Floc’h N, Louveau I, van Milgen J, Gondret F. Meal frequency changes the basal and time-course profiles of plasma nutrient concentrations and affects feed efficiency in young growing pigs, J. Anim. Sci. 2014, 92: 2008–2016. 10.2527/jas.2013-7505 24663180

[9] Newman RE, Downing JA, Thomson PC, Collins CL, Henman DJ, Wilkinson SJ. Insulin secretion, body composition and pig performance are altered by feeding pattern. Anim. Prod. Sci. 2014, 54: 319–328.

[10] Schneider JD, Tokach MD, Goodband RD, Nelssen JL, Dritz SS, DeRouchey JM, et al Effects of restricted feed intake on finishing pigs weighing between 68 and 114 kilograms fed twice or 6 times daily, J. Anim. Sci. 2011, 89: 3326–3333. 10.2527/jas.2010-3154 21934028

[11] Andretta I, Pomar C, Kipper M, Hauschild L, Rivest J. Feeding behavior of growing—finishing pigs reared under precision feeding strategies. J. Anim. Sci. 2016, 94: 3042–3050. 10.2527/jas.2016-0392 27482691

[12] Carcò G, Dalla Bona M, Carraro L, Latorre MA, Fondevila M, Gallo L et al Influence of mild feed restriction and mild reduction in dietary amino acid content on feeding behavior of group-housed growing pigs. Appl. Anim. Behav. Sci. 2018, 198: 27–35.

[13] Nielsen BL. On the interpretation of feeding behaviour measures and the use of feeding rate as an indicator of social constraint. Appl. Anim. Behav. Sci. 1999, 63: 79–91.

[14] Emmans GC, Kyriazakis I. Consequences of genetic changes in farm animals on food intake and feeding behaviour. Proc.Nutr. Soc. 2001, 60: 115–125. 11310416 10.1079/pns200059

[15] Emmans GC. A Method to predict the food intake of domestic animals from birth to maturity as a function of time. J. Theor. Biol. 1997, 186: 189–199.

[16] Nyachoti CM, Zijlstra RT, de Lange CFM., Patience JF. Voluntary feed intake in growing-finishing pigs: A review of the main determining factors and potential approaches for accurate predictions. Can. J. Anim. Sci. 2004, 84: 549–566.

[17] Schiavon S, Dalla Bona M, Carcò G, Carraro L, Bunger L, Gallo L. Effects of feed and indispensable amino acid restrictions on feed intake, growth performance and carcass characteristics of growing pigs. PLoS ONE 2018, 10.1371/journal.pone.0195645. PMC5886589 29621327

[18] Mordenti A, Bosi P, Corino C, Crovetto GM, Casa GD, Franci O, et al A methodological approach to assess nutrient requirements of heavy pigs in Italy. Ital. J. Anim. Sci. 2003, 2: 73–87.

[19] Ferguson NS, Arnold GA, Lavers G, Gous RM. The response of growing pigs to amino acids as influenced by environmental temperature. 1 Threonine. Anim. Sci. 2000, 70: 287–297.

[20] Schiavon S, Emmans GC. A model to predict water intake of a pig growing in a known environment on a known diet. Brit. J. Nutr. 2000, 84: 873–883. 11177204

[21] Topigs, 2012. Feeding manual Talent.https://varkens.nl/sites/default/files/Feeding%20Manual%20Talent%20progeny%202012.pdf. Accessed 05th April 2018.

[22] NRC. Nutrient Requirements of Swine. 11th revised ed Washington: National Academy Press; 2012.

[23] Kloareg M, Noblet J, Van Milgen J. Estimation of whole lipid mass in finishing pigs. Anim. Sci. 2006, 82: 241–251.

[24] EU. Commission Implementing Decision of 24 January 2014 authorising methods for grading pig carcases in Italy [notified under document C (2014) 279]. Off. J. L 23.

[25] EU. Corrigendum to Commission Implementing Decision 2014/38/EU of 24 January 2014 authorising methods for grading pig carcases in Italy (Off. J. L 23, 28.1.2014). Off. J. L 54.

[26] AOAC. Official Methods of Analysis of the Association of Official Agricultural Chemists, 19th ed AOAC International, 2012.

[27] SAS. SAS Institute, SAS/STAT. 9.4. Cary, NC. 2009

[28] de Haer LCM., Merks JWM. Patterns of daily food intake in growing pigs. Anim. Sci. 1992, 54: 95–104.

[29] Labroue F, Guéblez R, Sellier P. Genetic parameters of feeding behaviour and performance traits in group-housed Large White and French Landrace growing pigs. Genet. Sel. Evol. 1997, 29: 451–468.

[30] Hyun Y, Ellis M, Mckeith FK, Wilson ER. Feed intake pattern of group-housed growing-finishing pigs monitored using a computerized feed intake recording system. J. Anim. Sci. 1997, 75: 1443–1451. 9250503 10.2527/1997.7561443x

[31] Bornett HLI, Morgan CA, Lawrence AB, Mann J. The flexibility of feeding patterns in individually housed pigs. Anim. Sci. 2000, 40: 457–469. 10.1016/s0168-1591(00)00146-5 11080556

[32] Nielsen BL, Lawrence AB, Whittemore CT. Effect of group size on feeding behaviour, social behaviour, and performance of growing pigs using single-space feeders. Livest. Prod. Sci. 1995, 44: 73–85.

[33] Young RJ, Lawrence AB. Feeding behaviour of pigs in groups monitored by a computerized feeding system. Anim. Prod. 1994, 58: 145–152.

[34] von Felde A, Roehe R, Looft H, Kalm E. Genetic association between feed intake and feed intake behaviour at different stages of growth of group-housed boars. Livest. Prod. Sci. 1996, 47: 11–22.

[35] Schulze V, Roehe R, Bermejo JL, Looft H, Kalm E. The influence of feeding behaviour on feed intake curve parameters and performance traits of station-tested boars. Livest. Prod. Sci. 2003, 82: 105–116.

