# Supplementary material for: The influence of feeding behaviour on growth performance, carcass and meat characteristics of growing pigs
Source: PLoS One. 2018 Oct 15;13(10):e0205572. doi: 10.1371/journal.pone.0205572 (PMC6188860; doi:10.1371/journal.pone.0205572)
Supplement: S1 Table — (DOCX) [file pone.0205572.s001.docx]

**S1 Table. Partial correlations among feeding behavior traits (n = 92)^1^.**

| Item | Time spent eating | Feeding  visits | Feed intake  per visit | Feeding time  per visit | Feeding  rate |
| --- | --- | --- | --- | --- | --- |
| Feed intake | -0.143 | -0.003 | 0.203* | -0.190 | 0.506*** |
| Feeding time | - | 0.336** | -0.410*** | 0.226* | -0.892*** |
| Feeding visits |  | - | -0.808*** | -0.694*** | -0.245* |
| Feed intake per visit |  |  | - | 0.727*** | 0.394*** |
| Feeding time per visit |  |  |  | - | -0.318** |

^1^ *, **, and *** stand for P < 0.05, P < 0.01 and P < 0.001, respectively.
